# The Role of Glutathione and Sulfhydryl Groups in Cadmium Uptake by Cultures of the Rainbow Trout RTG-2 Cell Line

**DOI:** 10.3390/cells12232720

**Published:** 2023-11-27

**Authors:** Anke Lange, Helmut Segner

**Affiliations:** Department of Ecotoxicology, Helmholtz Center for Environmental Research UFZ, 04318 Leipzig, Germany

**Keywords:** GSH, fish cells, Cd uptake, sulfhydryl groups

## Abstract

The aim of this study is to investigate the role of cellular sulfhydryl and glutathione (GSH) status in cellular cadmium (Cd) accumulation using cultures of the rainbow trout cell line RTG-2. In a first set of experiments, the time course of Cd accumulation in RTG-2 cells exposed to a non-cytotoxic CdCl_2_ concentration (25 μM) was determined, as were the associated changes in the cellular sulfhydryl status. The cellular levels of total GSH, oxidized glutathione (GSSG), and cysteine were determined with fluorometric high-performance liquid chromatography (HPLC), and the intracellular Cd concentrations were determined with inductively coupled plasma mass spectrometry (ICP-MS). The Cd uptake during the first 24 h of exposure was linear before it approached a plateau at 48 h. The metal accumulation did not cause an alteration in cellular GSH, GSSG, or cysteine levels. In a second set of experiments, we examined whether the cellular sulfhydryl status modulates Cd accumulation. To this end, the following approaches were used: (a) untreated RTG-2 cells as controls, and (b) RTG-2 cells that were either depleted of GSH through pre-exposure to 1 mM L-buthionine-SR-sulfoximine (BSO), an inhibitor of glutathione synthesis, or the cellular sulfhydryl groups were blocked through treatment with 2.5 μM N-ethylmaleimide (NEM). Compared to the control cells, the cells depleted of intracellular GSH showed a 25% reduction in Cd accumulation. Likewise, the Cd accumulation was reduced by 25% in the RTG-2 cells with blocked sulfhydryl groups. However, the 25% decrease in cellular Cd accumulation in the sulfhydryl-manipulated cells was statistically not significantly different from the Cd accumulation in the control cells. The findings of this study suggest that the intracellular sulfhydryl and GSH status, in contrast to their importance for Cd toxicodynamics, is of limited importance for the toxicokinetics of Cd in fish cells.

## 1. Introduction

Heavy metals are common contaminants found in aquatic ecosystems [1]. With their ability to bioaccumulate and their intrinsic toxicity, heavy metals pose a risk to aquatic wildlife. The non-essential metal cadmium (Cd) shows particularly high toxicity to fish [2,3,4,5,6,7]. Cd can affect various molecular pathways and physiological systems, and therefore, the exposure of fish to Cd can result in a wide range of adverse effects, including the impairment of growth and development, the disruption of endocrine systems and osmoregulation, pathological changes in morphological and histological structures, or reduced stress tolerance (reviewed in [5,8]). These effects are often caused by the differential expression of genes responsible for various biological functions such as, for instance, oxidative stress, apoptosis, inflammation, or genotoxicity [9].

The uptake of Cd from a water body into fish depends on the metal speciation, with a free ion being the critical binding ligand [6,10,11], as well as on the cellular transport and uptake processes. To enter the cells of fish, metal ions have to pass across the lipid bilayer of cell membranes, which show low ion permeability. For essential metals, such as zinc (Zn) or copper (Cu), cells have evolved a variety of specific uptake mechanisms, including ion channels and carrier-mediated membrane transfer (active transport and/or facilitated diffusion) (e.g., [12,13,14]). In contrast, for non-essential metal ions, such as Cd, cells do not possess specific uptake mechanisms. To enter cells, Cd appears to rely, to a small extent, on diffusion, but mainly upon the hijacking of existing transport pathways for essential metals [15,16,17,18,19]. Particularly, calcium (Ca) channels appear to be utilized by Cd ions to cross the cell membrane, as has been demonstrated for several mammalian cell types [20,21], as well as for the chloride and gut cells of fish [22,23]. The hijacking of Ca transport mechanisms is possible since the hydrated ionic radii of Cd and Ca are very similar [24]. However, uptake pathways other than those that have evolved for Ca transport are also utilized by Cd. For instance, Liu et al. [25] showed that a Zn^2+^/[HCO_3_^−^]_2_ symporter can facilitate Cd uptake into cells; Ohta and Ohba [17] pointed to the involvement of divalent metal transporter 1 (DMT1) in Cd transport; and Oldham et al. [11] provided evidence for the role of zinc transporter 1. Finally, alternative to the absorption of Cd by transporters for inorganic metal species, Cd bound to sulfhydryl peptides can also be absorbed via membrane transport systems for mercaptides [14,26,27,28].

The vast majority of studies on cellular Cd uptake into fish cells have focused either on the role of changes in extracellular metal bioavailability for cellular metal accumulation or on the influence of the properties of ion transporters and ion channels on cellular Cd accumulation. An idea that has attracted less attention is the possible involvement of the intracellular sulfhydryl status in cellular Cd accumulation. The most abundant sulfhydryl-containing molecule in both prokaryotic and eukaryotic cells is glutathione (GSH). In most cell types, it is present at millimolar concentrations and hence constitutes more than 90% of the cellular non-protein sulfhydryl groups [29,30,31]. Other sulfhydryl-containing cellular molecules that can form complexes with metal ions include, for instance, metallothioneins but also amino acids such as cysteine [11]. Sulfhydryls have a high affinity for metal ions, which they efficiently capture by forming chelates [32]. As such, they are critical determinants for intracellular metal sequestration as well as for protecting cells against the toxic effects of metals. However, as outlined above, sulfhydryl molecules can also play a role in the membrane transport of Cd. The multiple roles of sulfhydryl-containing molecules in the cellular uptake, transport, and efflux of metals are likely to explain the findings of studies with mammalian cells that the sulfhydryl status influences cellular metal toxicokinetics (e.g., [14,33,34,35]). Whether such a toxicokinetic role of intracellular sulfhydryls is evolutionarily conserved among vertebrates and applies to other vertebrate groups such as teleost fish as well has, to the best of our knowledge, not been studied to date.

Isolated cell systems offer a number of advantages for acquiring toxicological information at the molecular and cellular levels [36,37]. In addition, they enable widely different species to be compared under equivalent exposure conditions for the cellular toxicokinetic and toxicodynamic processes of environmental contaminants and the evolutionary conservation or diversification of these processes to be unraveled [38,39]. Here, we use a fish cell line to explore whether intracellular sulfhydryl groups are involved in the uptake and accumulation of Cd in fish cells. Fish cell lines are frequently used models in environmental toxicology [39,40,41,42,43]. For the objectives of the present study, the use of an in vitro model enables the easy manipulation of the cellular sulfhydryl status as well as the sound control and analysis of external and internal metal concentrations. The model chosen for the purpose of the present study is the rainbow trout gonadal (RTG-2) cell line [44]. The RTG-2 cell line has been selected since it is the first immortal cell line established from fish in the 1960s, and RTG-2 is a long-established, stable, and well-characterized cell line that has repeatedly been used in studies on cellular metal metabolism [45,46,47]. Beyond this, we used this cell line in previous studies to evaluate the toxicodynamic role of GSH in Cd toxicity [48], which further supports the use of RTG-2 cells for the present toxicokinetic study. Here, we assessed the role of cellular sulfhydryl levels on Cd uptake in RTG-2 cells by comparing Cd accumulation in control cells and in cells with a manipulated sulfhydryl status. Modification of the sulfhydryl status was achieved through the pre-treatment of the cells with either N-ethylmaleimide (NEM) or with L-buthionine-SR-sulfoximine (BSO). NEM blocks the sulfhydryl group of not only GSH but also the sulfhydryl groups of proteins. BSO is a specific inhibitor of γ-glutamylcysteine synthetase [29], the rate-limiting enzyme in GSH synthesis [49]. The exposure concentrations of Cd, NEM, and BSO applied in the present experiments were not cytotoxic [48], this study.

## 2. Materials and Methods

### 2.1. Cell Culture

The rainbow trout gonad cell line RTG-2 [44] was obtained from the European Collection of Animal Cell Cultures (ECACC) as passage number 8. All cells used for this study were between passages 18 and 29. Cells were maintained routinely in 2-[4-(2-Hydroxyethyl)-1-piperazinyl]-ethanesulfonic acid (HEPES)-buffered Eagle‘s minimal essential medium (MEM) with Earle’s balanced salt solution, supplemented with 10% fetal calf serum, 2 mM of L-glutamine, 0.1% sodium bicarbonate, and 0.1 mg mL^−1^ of neomycin solution. RTG-2 cells were allowed to grow to confluence at 19 °C in 15 mL of the medium in disposable 75 cm^2^ culture flasks (Nunc) and then passaged several times until a sufficient stock was reached for the experiments. For subculturing, confluent cell monolayers were trypsinized using 0.05% (*w*/*v*) trypsin and 0.01% (*w*/*v*) EDTA in Ca^++^- and Mg^++^-free phosphate buffered saline (PBS), followed by a 3-fold dilution with medium. Except for L-glutamine and neomycin, which were obtained from Sigma-Aldrich (Taufkirchen, Germany), all reagents used for the cell culture were purchased from Biochrom (Berlin, Germany).

### 2.2. Cd Uptake

Cadmium uptake was measured in RTG-2 monolayers grown in 75 cm^2^ culture flasks. For the experiments, 15 mL of cell suspension with an initial density of 0.3 × 10^6^ cells mL^−1^ of culture medium were seeded into the culture flasks. After reaching semi-confluence, the used medium was aspirated from the cells. The cells were washed twice with Ca^++^- and Mg^++^-free PBS prior to the addition of a fresh culture medium containing 25 μM of CdCl_2_ (Merck, Darmstadt, Germany). The selected cadmium concentration corresponds to ≤20% of the cytotoxic concentration of CdCl_2_ in RTG-2 cells [48]. Ca^++^- and Mg^++^-free PBS was preferred to Ca^++^- and Mg^++^-containing PBS in order to avoid any effect on the membrane calcium channels and consequently on Cd uptake. Cells were exposed to a Cd-containing medium and incubated at 19 °C for up to 96 h. At the end of the exposure period, the cell viability was assessed using the neutral red (NR) assay [50] in order to verify that the experimental conditions were not cytotoxic to the RTG-2 cells. Exposure was terminated by removing the Cd-containing culture medium. The monolayers were quickly washed five times with ice-cold Ca^++^- and Mg^++^-free PBS. The first two of these washing steps were performed with PBS containing 2 mM of EDTA (Merck, Darmstadt, Germany) in order to extract the external labile metal fraction adsorbed to the cell surface. After washing, the cells were rapidly trypsinized, and after they had detached, the activity of trypsin was stopped through the addition of a culture medium. One mL of suspension was collected for a subsequent sulfhydryl analysis (total GSH, total cysteine, and GSSG) and protein determination (see below). The remaining cell suspension was centrifuged for 5 min at 50× *g* and 4 °C. After centrifugation, the supernatant was discarded, and the cells were resuspended in 1 mL of 65% HNO_3_ (Suprapure, Merck). The cells were digested in a water bath at 80 °C for 60 min before adding ultrapure water to a final volume of 10 mL. The medium, washing solutions, and centrifugation supernatant were kept and acidified through the addition of 65% HNO_3_ (Suprapure) to a final concentration of 1% (*v*/*v*). All samples were stored at 4 °C until the analysis.

The elemental Cd contents in the solutions were analyzed with inductively coupled plasma mass spectrometry (ICP-MS). The ICP mass spectrometer used for the determinations was a Perkin-Elmer ELAN 5000 equipped with an autosampler AS-90. To each sample, rhodium, at a final concentration of 0.1 μg mL^−1^, was added as the internal standard. Cd concentrations were quantified using calibration curves of 0–10 μg Cd L^−1^. The measurement of each sample was repeated 15 times.

The cell suspension taken for the sulfhydryl analysis and protein determination was spun for 5 min at 50× *g* and 4 °C, resuspended in 400 μL of 5 mM of diethylenetriaminepentaacetic acid (DTPA, Merck) dissolved in 0.12 N HCl, and then immediately frozen in liquid nitrogen. The samples were stored at −80 °C until the analysis. These samples were processed further for the sulfhydryl analysis and protein determination, as described below.

### 2.3. Cd Uptake under Different Intracellular Sulfhydryl Conditions

The influence of cellular sulfhydryl molecules on the Cd uptake rate was investigated by manipulating the levels of the cellular sulfhydryl groups or GSH, as described below.

#### 2.3.1. Effect of a Sulfhydryl Group Blocker on Cd Uptake

NEM (Sigma-Aldrich) was used as blocker of the sulfhydryl groups. For blocking, the cells were pre-exposed to 2.5 μM of NEM dissolved in culture medium for 30 min before being exposed to an extracellular Cd concentration of 25 μM of CdCl_2_ dissolved in an MEM medium containing 2.5 μM of NEM for 24 h. The chosen concentration of NEM was based on the results of the neutral red uptake cytotoxicity assay, which showed that 2.5 μM of NEM is not cytotoxic to RTG-2 cells (NR50_24h_ = 18.8 μM). The NEM-pre-exposed control cells remained in the NEM-containing culture medium without Cd.

#### 2.3.2. Effect of Depletion of Cellular GSH on Cd Uptake

For the depletion of GSH, the RTG-2 cells were pre-exposed to 1 mM of BSO (Sigma-Aldrich). In a previous study in our laboratory, the concentration of 1 mM of BSO was proven to be non-cytotoxic to RTG-2 cells [48]. After 24 h, the BSO-containing medium was replaced by a culture medium containing 25 μM of CdCl_2_. The control cells were pre-incubated with the BSO-containing medium, which was replaced by the MEM-containing medium without any addition of BSO after 24 h.

### 2.4. Determination of Total GSH, Total Cysteine and GSSG

The analytical detection of total GSH, total cysteine, and oxidized glutathione (GSSG) was carried out through high-performance liquid chromatography (HPLC), following a derivatization of the sulfhydryl groups with monobromobimane (mBBr), as previously described [51]. In brief, the samples were thawed on ice and homogenized through sonication for 20 s at 4 °C. The homogenates were centrifuged at 20,000× *g* and 4 °C for 30 min. The supernatant was used for the analysis of sulfhydryls, and the pellet was solubilized in 1 M of KOH for 60 min at 55 °C for protein determination.

The acid supernatant was separated into two aliquots for the determination of (a) total GSH (i.e., GSH + reduced GSSG) and total cysteine (i.e., cysteine + reduced cystine) and (b) for the analysis of GSSG, respectively. After neutralization of the extracts to a pH of 8.3 with a 2-(cyclohexylamino)ethanesulfonic acid buffer (CHES, Merck), pH 9.5, the disulfides in the aliquots for the determination of total GSH and total cysteine were reduced through the addition of 1,4-dithiothreitol (DTT, Merck). After incubation for 1 h at room temperature, the sulfhydryl groups in the reaction mixture were derivatized with mBBr (Molecular Probes; dissolved in acetonitrile) for 15 min in the dark. The reaction was stopped through the addition of 5% acetic acid.

Oxidized glutathione (GSSG) was determined as aliquots of reduced GSH, following the blocking of reduced sulfhydryl groups in the samples with NEM and the reduction of GSSG to GSH. For this, the second aliquots of the acid supernatant were neutralized, as described above. After the addition of NEM, the reaction was incubated for 10 min at room temperature before adding DTT. The subsequent steps, leading to the generation of bimane conjugates of GSSG, were as described above for the analysis of total GSH.

The bimane derivatives were separated on a reversed-phase LiChrospher 100 RP 18-column (5 μm; 4 × 250 mm, Merck) equipped with a guard column (5 μm; 4 × 4 mm) and integrated into a HPLC system 525, comprising an autosampler, a solvent degasser, a column oven (all Bio-Tek Instruments, Winooski, VT, USA), and a fluorescence detector (Jasco, Easton, MD, USA). Then, 20 μL of the sample was injected into the equilibrated column and separated at a constant column temperature of 25 °C and a flow rate of 1 mL min^−1^. The elution solvents were solution A, 10% methanol (HPLC grade, Merck), 0.25% acetic acid (Merck), and pH 3.9, and solution B, 90% methanol, 0.25% acetic acid, and pH 3.9. The elution profile was as follows: 0–3 min, 12% B; 3–4.5 min, 12–55% B; 4.5–14 min, 55% B; 14–17 min, 55–12% B; and 17–20 min, 12% B. The eluted derivatives were monitored fluorometrically at 380 nm (excitation) and 480 nm (emission).

Sulfhydryl concentrations were quantified through comparison with the standard curves of total GSH (0–172 nmol mL^−1^), GSSG (0–16 nmol mL^−1^), and L-cysteine (0–20 nmol mL^−1^) and expressed as nmol mg^−1^ protein. GSH and GSSG were purchased from Serva (Heidelberg, Germany); L-cysteine was obtained from Carl Roth (Karlsruhe, Germany).

For protein determination, the pellets from the acid-denatured cell homogenates were solubilized in KOH. The protein content was assessed using a detergent-compatible (DC) protein assay kit (Bio-Rad, Feldkirchen, Germany), based on the method of [52]. Bovine serum albumin (BSA, Serva) served as the standard protein. The measured protein contents were used to normalize both the intracellular Cd and sulfhydryl concentrations.

### 2.5. Statistical Analysis

Data are presented as the mean values ± standard deviation (SD) of two independent experiments with three to four replicates for each time point or treatment group, respectively, within each experiment. All data were normalized to 1 mg of protein, thus allowing a comparison of the results. The data were tested for normality and homogeneity of variances. In some cases, data were log-transformed prior to the tests to increase the homogeneity of their variances. Except where noted otherwise, statistical comparisons were performed with a one-way analysis of variance (ANOVA), followed by a multiple comparison using the Tukey test. Differences were considered significant at *p* < 0.05. Statistical analyses were carried out using SigmaStat 2.03 (Systat Software, Palo Alto, CA, USA).

## 3. Results

### 3.1. Analysis of Cd Concentrations in Exposure and Washing Media

The uptake experiments were performed using a nominal Cd concentration of 25 μM of CdCl_2_. The actual Cd content of the exposure medium was determined in each individual experiment at the beginning of the exposure in order to verify the concordance between the nominal and real exposure concentrations. The analytically determined mean Cd concentration in the media was 24.3 ± 3.6 μM Cd L^−1^ (*n* = 6), which is approximately 97% of the intended nominal concentration. This concentration amounts to ≤20% of the 24 h-NR50 concentration of Cd for RTG-2 cells, i.e., the concentration of Cd that reduces cell viability, as measured by the NR cytotoxicity assay, by 50% [48]. The cytotoxicity values in the actual experiments were monitored by the NR assay and in no case exceeded 20%. The differences in cell numbers between the control and exposed cells were accounted for by normalizing the Cd and sulfhydryl data to mg of protein.

To assess the Cd internalization from the exposure medium into the cells, it is essential to remove all extracellular Cd ions that are adsorbed to the cells. In the initial experiments, the conditions were established to quantitatively remove extracellularly adsorbed metal ions from the RTG-2 cells. To this end, the Cd-exposed cells were repeatedly washed with PBS + EDTA and/or PBS alone. The Cd contents were then analyzed in the aspirated culture medium as well as in eight subsequent wash solutions. The results of these experiments showed that externally bound Cd can be removed by washing the cells twice with PBS + EDTA, followed by three washing steps with PBS. After the fifth washing step, Cd was no longer detectable in the washing buffer. This washing procedure was used in the subsequent experiments so that the Cd contents measured in the RTG-2 cells after these washing steps could be assumed to truly represent intracellular Cd.

### 3.2. Time Course of Cadmium Uptake

**Figure 1:**
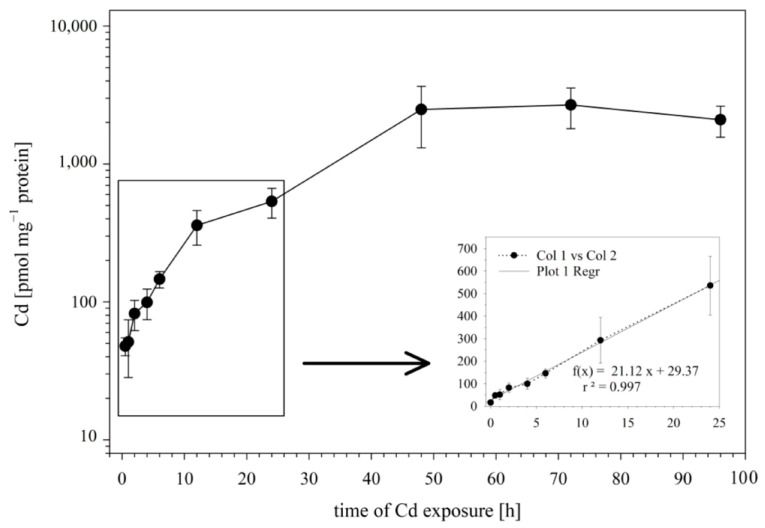
Cd uptake by RTG-2 cells as a function of time. Incubation of cells with 25 μM of CdCl_2_ started at t0. The control cells sampled before Cd addition showed Cd levels below the detection limit. The time course during the first 12 h is enlarged in the insert; the best-fit straight line of intracellular Cd accumulation is established using a linear regression model. Data represent mean values ± SD from two independent experiments, with three or four replicates for each time point within each experiment.

In the first set of experiments, we examined the influence of the incubation time on the Cd uptake by RTG-2 cells. To this end, the cells were incubated with 25 μM of Cd, and the Cd incorporation by the cells was analyzed after 0.5., 1, 2, 4, 6, 12, 24, 48, and 96 h of exposure. The time course of Cd uptake into the RTG-2 cells is shown in Figure 1. During the first 24 h of exposure, the Cd uptake followed a linear model, with an uptake rate constant of 21.12 pmol mg^−1^ protein h^−1^ (Figure 1 insert).

After the initial 24 h of exposure, a sustained slower uptake rate of Cd was observed until a plateau phase was reached at 48 h. At this time point, the cellular Cd concentrations no longer showed an increase but remained at a plateau level of 2431 ± 1048 pmol mg^−1^ protein.

### 3.3. Time Course of Cd Exposure Effects on Cellular Sulfhydryl Levels

**Figure 2:**
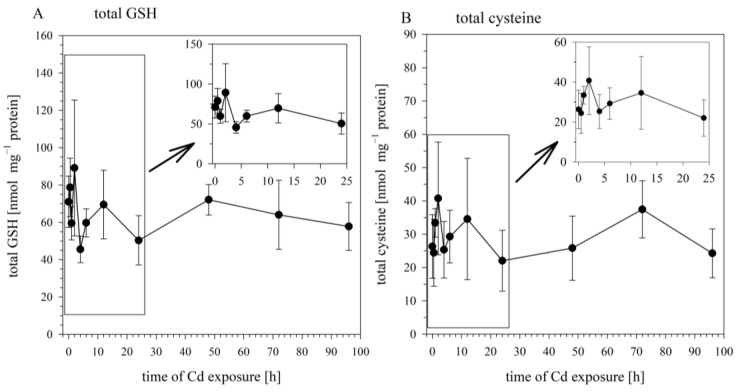
Time course of total GSH (**A**) and total cysteine (**B**) in RTG-2 cells exposed to 25 μM of CdCl_2_. Results represent mean values ± S.D. from two independent experiments with three to four replicates for each time point within each experiment. The time course during the first 24 h are enlarged in the insert. Cd did not exert significant effects on total GSH and total cysteine (*p* < 0.05).

When the cells were sampled for the analysis of intracellular Cd concentrations, aliquots from the same samples were taken and analyzed for the cellular concentrations of total GSH, total cysteine, and GSSG. The concentrations of these sulfhydryl-containing molecules in the Cd-exposed cells did not differ significantly from their concentrations in the non-exposed cells (at 0 h). The cellular levels of total GSH over the 24 h exposure period fluctuated around an average concentration of 67.3 ± 22.1 nmol mg^−1^ protein, with the concentrations at the different time points not significantly different (Figure 2A). The same goes for total cysteine, whose concentration varied around an average of 29.4 ± 13.5 nmol mg^−1^ protein (Figure 2B), and for GSSG, whose concentration varied around 1.23 ± 0.7 nmol mg^−1^ protein. Thus, the time-dependent increase in cellular Cd burdens over the 96 h exposure period was not associated with any significant alteration in the concentrations of the cellular sulfhydryl levels.

On the basis of the results of the time-course studies on Cd uptake and Cd-induced changes in cellular sulfhydryl levels, the 24 h time-point was selected as the standard sampling time for the subsequent experiments.

### 3.4. Effect of Blocking Cellular Sulfhydryl Groups on Cellular Cd Accumulation: NEM Experiments

To evaluate the role of the sulfhydryl groups in mediating cellular Cd uptake and accumulation, the RTG-2 cells were treated with NEM at a concentration of 2.5 μM during the 30 min before Cd exposure. Afterwards, the cells were co-exposed to NEM and 25 μM of CdCl_2_ for another 24 h.

**Figure 3:**
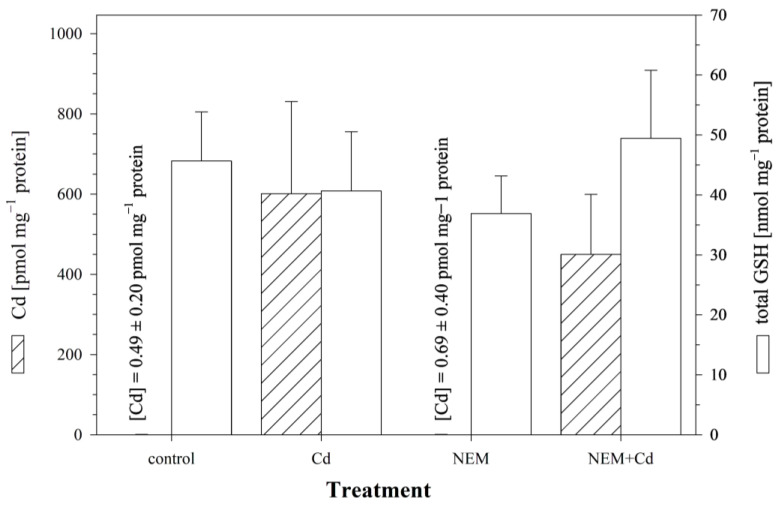
The effect of the sulfhydryl blocker NEM on intracellular Cd and total GSH levels in RTG-2 cells. Cells were exposed to 2.5 μM of NEM alone, 25 μM of Cd alone, or a combination of 2.5 μM of NEM and 25 μM of CdCl_2_. The exposure period was 24 h, following, in the case of the NEM treatments, a 30 min pre-incubation with 2.5 μM of NEM. Control cells were maintained under standard conditions for 24 h. The left *y*-axis displays intracellular Cd contents (hatched bars), and the right *y*-axis displays intracellular GSH levels (open bars). Each value represents the mean value ± standard deviation of two independent experiments, with four replicates for each treatment group within each experiment. No statistically significant differences could be observed (*p* < 0.05).

In the control cells, which were not exposed to NEM or Cd, only minute amounts of Cd (0.60 ± 0.35 pmol mg^−1^ protein) were detected after 24 h of incubation. When the cells were exposed to 25 μM of CdCl_2_ for 24 h, Cd accumulated in the cells, and the intracellular Cd levels reached a concentration of 601.2 ± 209.7 pmol mg^−1^ protein. The co-incubation of Cd with NEM reduced the level of intracellular Cd to 450.2 ± 138.2 pmol mg^−1^ protein (Figure 3), i.e., the cells co-treated with NEM and Cd accumulated only 75% of the Cd of the cells exposed to Cd alone. This reduction in Cd uptake, however, was not significant. In parallel to the measurement of cellular Cd levels, the same cells were also analyzed for the cellular concentrations of GSH. The results show that the intracellular Cd accumulation was not associated with a significant alteration in cellular GSH levels, neither in the cells exposed to Cd alone (40.7 ± 9.2 nmol GSH mg^−1^ protein) nor in the NEM-co-treated cells (49.5 ± 10.3 nmol GSH mg^−1^ protein) (Figure 3).

### 3.5. Effect of Manipulating Cellular GSH Levels on Cellular Cd Accumulation: BSO Experiments

**Figure 4:**
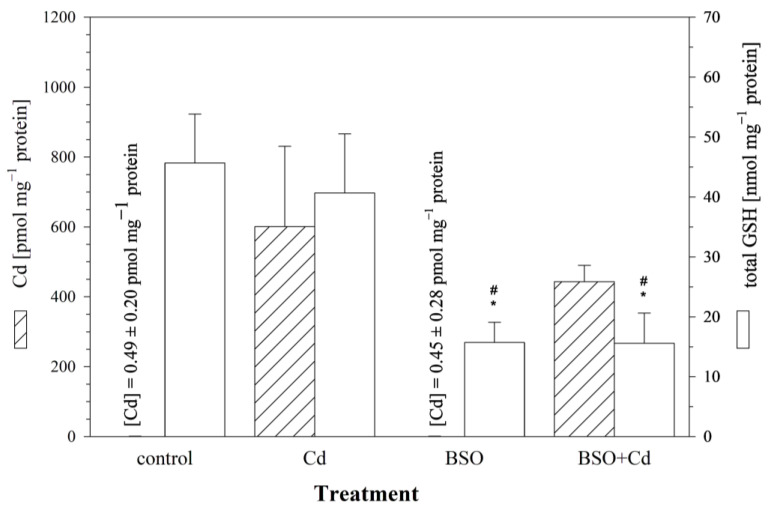
The effect of the γ-glutamylcysteine synthetase inhibitor BSO on cellular Cd and total GSH levels in RTG-2 cells. Control cells were maintained under standard conditions for 24 h. Cells were exposed to 25 μM of CdCl_2_ for 24 h without (Cd) and after 24 h pre-incubation with 1 mM of BSO (BSO + Cd). The left *y*-axis displays intracellular Cd contents (hatched bars), and the right *y*-axis displays intracellular GSH levels (open bars). Data represent mean values ± standard deviation of two independent experiments, with four replicates for each treatment group within each experiment. * indicates statistically significant different compared to the untreated RTG-2 cells (*p* < 0.05); # denotes statistical significant difference versus cells exposed to Cd alone (*p* < 0.05).

To explore the influence of intracellular GSH levels on Cd uptake, the cells were pre-incubated with BSO in order to reduce the cellular GSH levels. After 24 h, the culture medium was changed, and the cells were incubated for another 24 h with (i) the medium, (ii) 25 μM of Cd, (iii) 1 mM of BSO, or (iv) BSO + Cd. The measured intracellular GSH level after a 24 h pre-exposure of the RTG-2 cells to 1 mM of BSO was 45.7 ± 7.6 nmol mg^−1^ protein, corresponding to a significant reduction of 70% compared to the control cells. Only minute amounts of Cd (0.45 ± 0.28 pmol mg^−1^ protein) were detected in the cells incubated without Cd. The RTG-2 cells with reduced GSH levels due to BSO treatment showed a decreased uptake of Cd compared to the control cells (Figure 4). After a 24 h exposure to 25 μM of Cd, only 74% (443.4 ± 43.1 pmol Cd mg^−1^ protein) of the Cd was taken up in the GSH-reduced cells compared to 601.2 ± 209.7 pmol Cd mg^−1^ protein in the Cd-exposed control cells (Figure 4). This difference between the GSH-reduced and control cells, however, was not significant.

In parallel to the measurement of cellular Cd levels, the cells were also analyzed for cellular concentrations of total GSH. The 24 h exposure to 25 μM of Cd had no additional effect on the cellular GSH levels in the GSH-reduced cells (15.6 ± 4.8 nmol GSH mg^−1^ protein) compared to the GSH-depleted control cells (15.7 ± 3.2 nmol GSH mg^−1^ protein) (Figure 4).

### 3.6. Intracellular Molar Ratio of Cd to GSH in RTG-2 Cells with Differently Modulated Sulfhydryl Status

**Figure 5:**
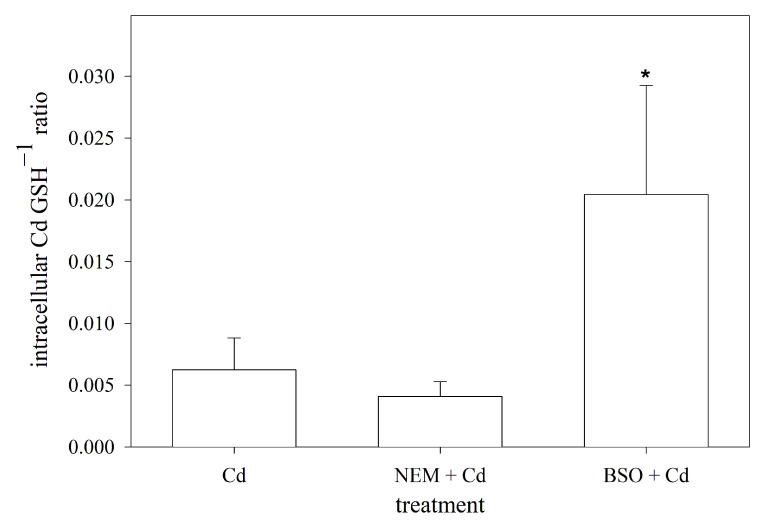
Intracellular molar ratio of Cd to GSH in RTG-2 cells with differently modulated sulfhydryl status. Cells were exposed to 25 μM of CdCl_2_ for 24 h, and Cd levels were subsequently measured, under control conditions, in cells co-incubated with 2.5 μM of NEM and 25 μM of CdCl_2_ for 24 h after 30 min pre-incubation with 2.5 μM of NEM (NEM + Cd) or after 24 h pre-incubation with 1 mM of BSO (BSO + Cd). Data represent mean values ± standard deviation of two independent experiments with four replicates within each experiment. * indicates statistically significant difference compared to Cd-control RTG-2 cells (*p* < 0.05).

When exposed to Cd, the primary and most prevalent complex formed with GSH is Cd(GS)_2_ [53]. Assuming that two GSH molecules are required to sequester one Cd ion in RTG-2 cells, the cellular ratio of Cd to GSH was calculated using the measured intracellular concentrations of both. By doing so, the data showed that the blocking of the sulfhydryl groups by NEM did not affect the intracellular Cd:GSH ratio compared to the non-manipulated cells. The depletion of the intracellular GSH levels, on the other hand, resulted in a significantly higher cellular Cd:GSH ratio compared to the normal RTG-2 cells (Figure 5).

## 4. Discussion

The aim of the present study was to assess the role of sulfhydryl groups, including the most abundant low-molecular-weight sulfhydryl molecule, GSH, in Cd accumulation in the fish cell line RTG-2. To this end, we initially identified some appropriate exposure conditions. This was the purpose of the time-course experiment. The findings from the time-course experiment show that the intracellular Cd concentrations, after an initial rapid increase, approach a plateau level within 24 to 48 hrs after the start of the exposure. Apparently, within this time period, the RTG-2 cells could establish an equilibrium between Cd influx, sequestration, and efflux. Therefore, we considered a 24 h exposure duration to be suitable for assessing whether the cellular sulfhydryl status influences the level of Cd accumulation in RTG-2 cells. An additional advantage of this time window is that metallothionein (MT) mRNA levels are hardly detectable in RTG-2 cells after 24 h of Cd exposure [47], indicating that the cells do not yet display a relevant upregulation of MT protein synthesis. The different MT protein levels between the control and exposed cells could have confounded the comparability of the controls versus the treatments. The levels of other sulfhydryl-containing molecules that can bind Cd ions such as amino acids of the culture media were also identical among the various treatments.

A further important insight obtained from the initial time-course experiment is that the Cd exposure concentration in the present study did not modulate the sulfhydryl levels in the RTG-2 cells. A number of studies have observed that Cd exposure can result in reduced tissue concentrations of GSH and other sulfhydryls [54,55]. In particular, higher Cd concentrations appear to be associated with reduced GSH levels, while lower concentrations remain without effect or may even cause a transitory increase [51,55]. For the comparability of the different treatments in our study, it was important that the intracellular sulfhydryl levels are only modulated by the BSO and NEM treatments, not by the Cd exposure itself. The outcome of the time-course experiments confirms this assumption. An important side observation of the analysis of the time-dependent Cd and sulfhydryl changes is that, on a molar basis, the intracellular Cd concentrations remained constantly lower than the intracellular GSH concentrations. This suggests that the Cd treatment did not (over)saturate the GSH pool of the RTG-2 cells but the pool quantitatively should have been sufficient to sequester all entering Cd ions.

Once we had identified, by means of the initial experiments, the appropriate exposure conditions, we started a second set of experiments in which we examined the influence of the sulfhydryl groups on the cellular Cd levels. Intracellular metal levels are mainly determined by three processes, which are metal influx, metal sequestration, and metal efflux [18,56,57,58]. All three processes are potentially influenced by cellular sulfhydryl contents. The influx involves the initial association of the metal with the membrane, followed by a sustained transfer across the cell membrane (e.g., [59]), and is mediated by diverse membrane transporters and channels. Further processes, like the diffusion or transfer of metals by GSH transporters, appear to play a role as well [14,26,27,60,61]. Once the metal has reached the cytoplasm, it is efficiently sequestrated to protect the cell against the toxic action of the free metal ions. The key drivers for intracellular metal sequestration and storage are the sulfhydryl groups. In fact, in most cell types, the levels of available sulfhydryl compounds are essential in protecting cells against metal toxicity (e.g., [48,62,63]). Finally, sulfhydryl–metal complexes are also involved in the efflux of metals from cells [26,57].

Most studies on the interaction between sulfhydryl-containing molecules and Cd have focused on the cytoprotective role of sulfhydryls [5,64]. In contrast, the present study investigated the possible role of sulfhydryl-containing molecules in the cellular toxicokinetics of Cd. Methodologically, we approached this question by manipulating the sulfhydryl status of the RTG-2 cells. The experimental results indicate that the reduced intracellular availability of sulfhydryl groups, be it because of BSO-induced inhibition of GSH synthesis or because of the NEM-induced blockage of sulfhydryl groups, leads to reduced Cd accumulation in RTG-2 cells. Despite the different modes of action of the two sulfhydryl-modifying agents, the Cd accumulation was lowered by the same order of magnitude (25%) when using both approaches. Whether this implies that both agents affect the same mechanism or that this is an arbitrary coincidence cannot be answered from the data of the present study.

Although the effect of manipulating the cellular sulfhydryl status on the Cd accumulation in the RTG-2 cells was not significant, the trend for reduced Cd accumulation in the sulfhydryl-depleted cells agrees with observations from the literature. Decreases in the range of 21 to 33% in the uptake of Cd were reported for NEM-treated rat hepatocytes [33] and the human hepatic cell line WRL-68 [61]. In contrast, up to a 90% reduction in Cd transport was observed for NEM-treated Caco-2 cells [14]. With respect to BSO, there appear to exist no published in vitro or in vivo studies on its influence on cellular Cd accumulation, but for mercury, the accumulation in rat kidneys was reduced by about 50% in BSO-pre-treated animals. The reduced renal mercury accumulation in BSO-treated rats occurred in concordance with lowered renal GSH levels [65]. Similarly, the uptake of Cu into mammalian HEK293 cells was reduced by 50% when the cells were pre-exposed to BSO [34]. Thus, the available literature data as well as the findings from the present study all point to the role of the cellular sulfhydryl status on metal toxicokinetics, although quantitatively, the influence appears to be moderate.

Several mechanisms may underlie the involvement of the cellular sulfhydryl status in Cd accumulation. One possible mechanism could be the blockage or reduction in sulfhydryl-containing molecules in the cell membrane, which are known to act as anchors for binding and transporting Cd. In rat hepatocytes and rat renal cortical epithelial cells, the binding of Cd to plasma membrane sulfhydryl groups is an essential step in the transport of Cd in these systems [18]. Therefore, as suggested by [14], the membrane sulfhydryl groups could act as an anchor for the binding of Cd ions. By blocking these reactive groups in the cell membranes, the uptake of Cd into the cells would be reduced. Another possible mechanism is an effect of intracellular GSH on the metal concentration gradient over the membrane, with intracellular GSH maintaining the gradient by forming complexes with metal ions entering the membrane. This mechanism might be an explanation for the similarity in the magnitude of the BSO and NEM effects on the Cd uptake in RTG-2 cells, since in this case, the same cellular parameter—the intracellular GSH pool—would be the responsible mechanism. It is also conceivable that Cd uptake rates are determined by intracellular Cd/GSH ratios, thus representing another possibility through which sulfhydryls could have an influence on cellular Cd accumulation. Finally, the possible role of sulfhydryl-containing molecules in the cellular Cd efflux should not be missed, as it could be of direct relevance for the intracellular equilibrium concentration of Cd [26,57]. Future studies may attempt to identify the role of the different cellular compartments in the metal–sulfhydryl interaction by using microscopical techniques. High-resolution microscopy, particularly when using labeled probes, enables the transfer of metals into and inside cells to be followed [66,67,68] and may help visualize how a change in the sulfhydryl status changes the cellular fate of metal ions.

## 5. Conclusions

In conclusion, the findings of the present study provide evidence that sulfhydryl-mediated components are involved, to a limited extent, in the Cd accumulation of RTG-2 cells. Thus, in addition to their well-documented influence on Cd toxicodynamics in fish cells, sulfhydryl groups appear to be involved in the toxicokinetics of Cd in fish cells.

## Data Availability

The data presented in this study are available on request from the corresponding author.

## Abbreviations

ANOVA—analysis of variance; BSA—bovine serum albumin; BSO—buthionine-SR-sulfoximine; Ca—calcium; Cd—cadmium; CHES—2-(cyclohexylamino)ethanesulfonic acid buffer; Cu—copper; DMT1—divalent metal transporter 1; DTPA—diethylenetriaminepentaacetic acid; ECACC—European Collection of Animal Cell Cultures; GSH—glutathione; GSSG—oxidized glutathione; HEPES—2-[4-(2-Hydroxyethyl)-1-piperazinyl]-ethanesulfonic acid; HPLC—high-performance liquid chromatography; ICP-MS—inductively coupled plasma mass spectrometry; mBBr—monobromobimane; MEM—minimal essential medium; MT—metallothionein; NEM—N-ethylmaleimide; NR—neutral red; PBS—phosphate buffered saline; RTG-2—rainbow trout gonad-2; SD—standard deviation; Zn—zinc.
